# Effect of Preoperative Anesthesia Consultation on Decreasing Anxiety in Patients Undergoing Oral and Maxillofacial Surgery

**DOI:** 10.30476/DENTJODS.2019.77883.0

**Published:** 2020-06

**Authors:** Fahimeh Akhlaghi, Shahabaldin Azizi, Bahman Malek, Farinaz Mahboubi, Shahin Shams, Mahdieh Karimizadeh

**Affiliations:** 1 Dental Research Center, Research Institute of Dental Sciences, Dental School, Dept. of Oral and Maxillofacial Surgery, Shahid Beheshti University of Medical Sciences, Tehran, Iran; 2 Resident of Oral and Maxillofacial Surgery, Taleghani Hospital, Shahid Beheshti University of Medical Sciences, Tehran, Iran; 3 Dept. of Anesthesiology, Taleghani Hospital, Shahid Beheshti University of Medical Sciences, Tehran, Iran; 4 Power Electrical Engineer, Tehran University, Tehran, Iran; 5 Oral and Maxillofacial Surgeon, Tehran, Iran; 6 Pediatrician, Khalij Hospital, Bushehr University of Medial Sciences, Bushehr, Iran

**Keywords:** Anxiety, Maxillofacial Surgery, Oral Surgery, Preoperative Consultation

## Abstract

**Statement of the Problem:**

Preoperative anxiety is the subject of major concern for many patients.

**Purpose:**

The current study aimed at determining the effect of anesthesia consultation on decreasing anxiety in patients undergoing oral and maxillofacial surgery.

**Materials and Method:**

This randomized clinical trial was conducted on 250 patients undergoing different maxillofacial surgeries. The data collection instruments included a questionnaire containing the Spielberger state-trait anxiety inventory (STAI) and a researcher-made questionnaire with queries on the demographic characteristics and surgery-related information. Analysis of the data was performed in SPSS, using descriptive and inferential statistics.

**Results:**

The findings showed that the majority of patients (38.4%) had moderate anxiety; there was no significant difference between the consultation and control groups in terms of age and gender. Also, the scores of state and trait anxiety were significantly lower in the consultation group, compared with the control group.

**Conclusion:**

The present results showed that preoperative anesthetic consultation reduced preoperative anxiety, compared with the control group. Our findings suggest that anesthetic counseling services should be provided for individuals experiencing high levels of stress.

## Introduction

Preoperative anxiety is widely accepted as an expected response of patients who are candidates for surgery [ 1 - 2 ]. It begins as soon as the surgical procedure is planned and reaches its maximum intensity upon entry to the hospital [ 3 ]. It is described as an unpleasant emotional experience, which involves feelings of tension, apprehension, nervousness, high autonomic activity (sympathetic and parasympathetic) and endocrine stimulation. It may lead to the patient’s failure to attend the planned surgical procedure [ 4 ].

Preoperative anxiety has been reported in 11-80% of adult patients [ 5 ]. Accordingly, there has been growing interest in the study of anxiety-reducing interventions and the possible effects of preoperative anxiety on the course and outcomes of surgical treatments [ 6 ]. Although many studies have been conducted on medications used to relieve preoperative anxiety, still little is known about the incidence or etiology of this phenomenon [ 5 - 6 ]. This type of anxiety seems to be directly related to the fear of an unfamiliar environment, loss of control, and fear of death and disfigurement. Generally, management and reduction of anxiety can be challenging, as it may complicate anesthesia induction and alter the pharmacokinetics of therapeutic agents by inducing catecholamine release [ 7 ].

Today, there are two main approaches to reduce the patients’ anxiety. First, a preoperative visit is necessary for reassurance and encouragement of patients in order to reduce anxiety, shorten the length of hospital stay, and reduce the need for narcotic drugs. The second approach is prescribing pharmacological anxiolytic medications such as benzodiazepines or narcotics [ 8 ]. Overall, preoperative anxiety and fear can complicate the management of anesthesia and the postoperative period. Considering the importance of anxiety in patients undergoing surgery, its prevention is essential. Therefore, the current randomized clinical trial (RCT) was conducted to determine the effects of anesthesia consultation on anxiety in patients undergoing oral maxillofacial surgeries.

## Materials and Method

This RCT was conducted on patients undergoing different maxillofacial surgeries, admitted to Taleghani Educational Hospital, affiliated to Shahid Beheshti University of Medical Sciences, Tehran, Iran. Sedative premedication was not used for patients participating in the study. The study objectives were explained to the participants, and formal consent was obtained for participation. It was affirmed that contribution to the study is completely voluntary and that the data are kept confidential. The study protocol was approved by the Ethics Committee of Research Institute of Dental Sciences, Dental School of Shahid Beheshti University of Medical Sciences (ethical code: IR.SBMU.DRC. REC.1392.11).

Considering the 95% confidence interval, 80% statistical power, and an estimated 25% of moving frequency in the cycle change of the consultation group, the sample size was calculated to be 250. The inclusion criteria were as follows: 1) age ≥ 12 years; 2) American Society of Anesthesiology (ASA) physical status I to III; 3) ability to give an informed consent; and 4) having no pain during data collection. On the other hand, the exclusion criteria were as follows: 1) having a malignancy; 2) impaired cognitive function; 3) age ˂ 12 years; 4) need for emergency surgery; and 5) history of chronic diseases.

At the beginning of the study, the participants were categorized into four operation groups: trauma for more than one month (n= 8); trauma for less than one month (n=50); non-traumatic/non-esthetic (n= 62); and esthetic (n= 130). Next, the participants were randomly divided into two groups of consultation (n=125) and control (n=125). The night before surgery and receiving any sedatives, the anesthesiology resident informed the patients in the consultation group about the details of anesthesia and surgical procedures.

For data collection, the Spielberger state-trait anxiety inventory (STAI), which shows high validity and reliability for the assessment of state and trait anxiety, was used to determine the anxiety intensity. This inventory consisted of 40 questions translated into Farsi. The reliability (r=0.97) and validity of the translated version have been reported in Persian journals [ 9 ]. The total score of STAI ranges from 40 as no anxiety to 160 as maximum anxiety [ 10 ].

In addition, a researcher-made questionnaire, consisting of two parts, was used to assess the patients’ anxiety level. The first part included demographic data, such as gender, age, and level of education, while the second part consisted of 40 questions, based on the Spielberger STAI. It should be mentioned that STAI has been used in many studies [ 11 - 13 ] and its mean reliability has been estimated at 97%; therefore, there was no need to determine its reliability in the present study.

All analyses were performed in SPSS version 18. The level of significance was considered to be less than 0.05.

## Results

The current study was conducted on 250 subjects, including 133 females (53.2%) and 117 males (46.8%). The mean age of the subjects was 27 years. The distribution of patients in the operation groups is shown in Table 1. There was no significant difference between the consultation and control groups in terms of age and gender. Also, the scores of state and trait anxiety were significantly lower in the consultation group, compared with the control group, as shown in Table 2.

**Table 1:** Distribution of patients in the consultation group

Trauma˃ 1m, N (%)	Trauma ˂ 1m, N (%)	Non-traumatic/ non-static, N(%)	Non-traumatic /static, N (%)
8 (3.2)	50 (20)	62 (24.8)	130 (52)

**Table 2:** Demographic and clinical characteristics of the study groups

Characteristics	Consultation (N= 125)	Control (N= 125)	*p* Value
Age (years)	28.9±11.1	29.9±11.8	0.51
Gender ratio (M/F)	54/71	63/62	0.25
Y1 score	32.2±12.0	46.0±10.9	<0.001
State anxiety, n (%)	19 (15.2)	70 (56.0)	<0.001
Y2 score	41.4±11.9	41.8±9.2	<0.001
Trait anxiety, n (%)	63 (16.0)	65 (52.0)	<0.001

**Figure 1:**
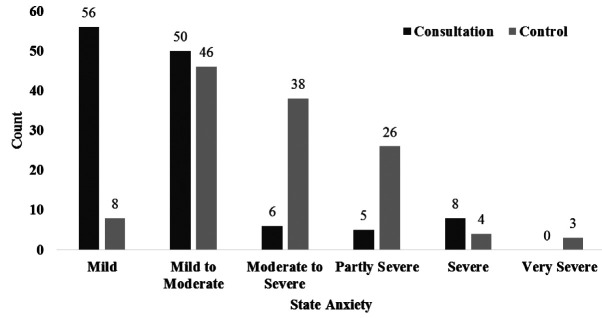
The anxiety scores of the groups based on STAI

The results also showed that 38.4% of subjects had moderate anxiety, as shown in Figure 1.

## Discussion

Anxiety is one of the most common problems of patients prior to surgery. Although the available surgical techniques are quite advanced, most patients still experi-ence anxiety before surgery. Currently, modalities, such as preoperative premedication and psychological preparation programs, are being used to treat preoperat-ive anxiety in adult patients. The current RCT aimed at evaluating the effect of preoperational anesthesia consu-ltation on decreasing anxiety in patients undergoing oral and maxillofacial surgeries using the Spielberger STAI.

The present results demonstrated that patients undergoing anesthesia consultation had less preoperative anxiety, compared to the control group. Overall, it is difficult to compare the results of the present study with those of previous studies regarding preoperative anxiety due to differences in their methodology, study population, and study design. Egbert *et al*. [ 14 ] concluded that the anesthetic visit was effective in reducing preoperative anxiety, which is almost similar to the present study. They also studied anxiety in inpatients prior to elective surgery. On the contrary, Twersky *et al*. [ 15 ], using the same method as the present study for anxiety assessment, stated that the patients did not experience a reduction in preoperative anxiety by visiting the anesthesiologist before surgery. In another study, Leigh *et al*. [ 16 ] evaluated the effects of anesthetic visits on anxiety using STAI and found a reduction in the anxiety scores of patients, who were assessed preoperatively by the anesthesiologist.

## Conclusion

In the present study, anesthetic consultation reduced preoperative anxiety in the consultation group, compared with the control group. The present results suggest that counseling services should be provided for individuals experiencing high levels of stress. In addition, our findings may be useful in various aspects of patient care, including education of medical students, treatment, and research.

## References

[1] Norris W, Baird WL ( 1967). Pre-operative anxiety: a study of the incidence and aetiology. Br J Anaesth.

[2] Johnston M ( 1980). Anxiety in surgical patients. Psychol Med.

[3] Williams JG, Jones JR ( 1968). Psychophysiological responses to anesthesia and operation. JAMA.

[4] Erkilic E, Kesimci E, Soykut C, Doger C, Gumus T, Kanbak O ( 2017). Factors associated with preoperative anxiety levels of Turkish surgical patients: from a single center in Ankara. Patient Prefer Adherence.

[5] Caumo W, Schmidt AP, Schneider CN, Bergmann J, Iwamoto CW, Bandeira D ( 2001). Risk factors for preoperative anxiety in adults. Acta Anaesthesiol Scand.

[6] Wiens AG ( 1998). Preoperative anxiety in women. AORN J.

[7] Woldegerima Y, Fitwi G, Yimer H, Hailekiros A ( 2018). Prevalence and factors associated with preoperative anxiety among elective surgical patients at University of Gondar Hospital. Gondar, Northwest Ethiopia, 2017. A cross-sectional study. International Journal of Surgery Open.

[8] Hermes D, Matthes M, Saka B ( 2007). Treatment anxiety in oral and maxillofacial surgery. Results of a German multicentre trial. J Craniomaxillofac Surg.

[9] Sahebi A, Asghari MJ, Salari RS ( 2015). Validation of depression anexity and stress scale (DAAS-21) for an Iranian population. Journal of Iranian Psychologists.

[10] Spielberger CD ( 1970). State‐Trait anxiety inventory. The Corsini encyclopedia of psychology.

[11] Csemiczky G, Landgren BM, Collins A ( 2000). The influence of stress and state anxiety on the outcome of IVF-treatment: psychological and endocrinological assessment of Swedish women entering IVF-treatment. Acta Obstet Gynecol Scand.

[12] Gregurek R, Pavic L, Vuger-Kovacic D, Vukusic H, Potrebica S, Bitar Z ( 2001). Increase of frequency of post-traumatic stress disorder in disabled war veterans during prolonged stay in a rehabilitation hospital. Croat Med J.

[13] Kalkhoran MA, Karimollahi M ( 2007). Religiousness and preoperative anxiety: a correlational study. Ann Gen Psychiatry.

[14] Egbert LD, Battit G, Turndorf H, Beecher HK ( JAMA 1963). The value of the preoperative visit by an anesthetist. A study of doctor-patient rapport.

[15] Twersky RS, Lebovits AH, Lewis M, Frank D ( 1992). Early anesthesia evaluation of the ambulatory surgical patient: does it really help?. J Clin Anesth.

[16] Leigh JM, Walker J, Janaganathan P ( 1977). Effect of preoperative anaesthetic visit on anxiety. Br Med J.

